# The Plant-Derived Chalcone 2,2′,5′-Trihydroxychalcone Provides Neuroprotection against Toll-Like Receptor 4 Triggered Inflammation in Microglia

**DOI:** 10.1155/2016/6301712

**Published:** 2015-12-21

**Authors:** Manasi Jiwrajka, Alexandra Phillips, Matt Butler, Miriam Rossi, Jennifer M. Pocock

**Affiliations:** ^1^Mayne Medical School, 288 Herston Road, Brisbane, QLD 4006, Australia; ^2^Department of Neuroinflammation, University College London Institute of Neurology, 1 Wakefield Street, London WC1N 1PK, UK; ^3^Department of Chemistry, Vassar College, 124 Raymond Avenue, Poughkeepsie, NY 12604-0484, USA

## Abstract

Chalcones are plant metabolites with potential for therapeutic exploitation as antioxidant, anti-inflammatory, and antiproliferative agents. Here we explored the neuroprotective effects of 2,2′,5′-trihydroxychalcone (225THC), a potent antioxidant with radical-scavenging properties. 225THC was found to be a potent inhibitor of apoptosis in stimulated primary rat neuronal cultures. This was likely mediated by an anti-inflammatory effect on microglial cells since 225THC inhibited LPS-stimulated TNF-*α* and IL-6 secretion from primary rat microglia and modulated the cytokine/chemokine profile of BV2 microglial cells. Additionally, 225THC inhibited LPS-evoked inducible nitric oxide synthase expression but did not influence endogenous superoxide generation. Microglial flow cytometric analyses indicated the 225THC treatment induced a shift from an M1-like phenotype to a more downregulated microglial profile. Taken together these data suggest that the chalcone 2,2′,5′-trihydroxychalcone can modulate neuroinflammatory activation in brain-derived microglia and holds promise as a therapeutic in neuroinflammatory conditions.

## 1. Introduction

Plants produce secondary metabolites that protect them from toxins and insects. Some of these plant metabolites, such as chalcones, have significant antioxidant, anti-inflammatory, and antiproliferative properties in a range of cell types [1–7]. Chalcones are similar to other known antioxidants such as resveratrol, curcumin, and ubiquinone and are the natural precursors of flavonoids and isoflavonoids in higher plants [4, 8–10]. In plants, chalcones protect against UV exposure, pathogens, and insects, and their antioxidant and anti-inflammatory properties make them of increasing interest in the treatment of human conditions such as cancer, inflammation, tuberculosis, and malaria [2, 7, 11].

Stress and injury to cells can cause the production of free radicals and the release of cytokines. In the brain, such substances are produced by the activation of microglia, the brain's resident phagocytes, leading to neurotoxicity [12–14]. During ageing, neurodegeneration, ischaemia, brain injury, or other neuropathologies, there is enhanced production of free radicals and cytokines, increased apoptosis, and reduced expression of synaptic or growth proteins [15–18].

In the brain, the chalcone isoliquiritigenin has anxiolytic effects [19] whilst two chalconoids from the desert plant* Pulicaria incisa* inhibited the production of reactive oxygen species (ROS) by astrocytes and prevented their oxidant-induced cell death [20]. One plant-derived chalcone, 2,2′,5′-trihydroxychalcone (225THC), demonstrated strong antioxidant and radical-scavenging properties in L-6 myoblasts and THP-1 human monocytes [21]. However, the neuroprotective effects of this particular chalcone on cells of the CNS are unknown and the subject of the present study.

## 2. Materials and Methods

### 2.1. Cell Culture

#### 2.1.1. BV2 Microglia

The BV2 mouse microglial cell line was a kind gift from Dr. Claudie Hooper, Institute of Psychiatry, Kings College London, and was originally obtained from the Department of Life Sciences, National Cheng Kung University, Taiwan. The cells were cultured in RPMI-1640 medium (Gibco, Life Technologies) plus 5% foetal bovine serum (FBS) and 50 U/mL penicillin and 50 *μ*g/mL streptomycin (all Invitrogen, http://www.invitrogen.com/) at 37°C, at 5% CO_2_, and at a density of 2 × 10^4^ per 13 mm glass coverslips in 24-well plates or 1 × 10^5^ per 6-well plate for Western blotting or FACS analyses and rested overnight at 37°C plus 5% CO_2_. Before use, cell medium was replaced with RPMI plus 1% FBS.

#### 2.1.2. Primary Cultures of Microglia and Cerebellar Granule Cell Neurons

Sprague Dawley rat pups (postnatal day 5) were bred and reared in-house from stock animals (Charles River UK, Kent, UK) and were sacrificed in accordance with Schedule 1 of the Animals Scientific Procedures Act (1986) UK, for the culture of primary microglia and cerebellar neurons. Microglia were cultured as described previously [22]. Briefly, cells were plated on 13 mm glass coverslips in 24 well plates at 0.5 × 10^5^ cells/well and maintained in culture medium consisting of MEM (Invitrogen, http://www.invitrogen.com/), supplemented with 10% FBS, 20 mM KCl, 20 mM D-glucose, 2 mM D-glucose, 25 mM NaHCO_4_, 50 U/mL penicillin, 50 *μ*g/mL streptomycin, and 6 *μ*g/mL ampicillin. The microglia were maintained at 37°C, 6% CO_2_ and used 1 day after plating. Where possible experiments were carried out using primary microglia but where assays required higher cell numbers, BV2 microglia were used. We have previously compared responses of BV2 microglia and primary microglia and have found not obvious differences in responses. Cerebellar granule cells (CGCs) were cultured as described previously [22]. The cells were plated on 13 mm glass coverslips in 24 well plates at a density of 8 × 10^5^/coverslip and maintained in culture medium (MEM plus 10% FBS, 20 mM KCl, 30 mM D-glucose, 2 mM L-glutamine, 25 mM NaHCO_4_, 50 U/mL penicillin, 50 *μ*M streptomycin, and 6 *μ*g/mL ampicillin) that was later supplemented with 20 *μ*M cytosine furanoarabinoside (Ara-C) to control glial proliferation. The cells were maintained at 37°C, 6% CO_2_ and used after 8 days* in vitro* (8 DIV).

### 2.2. Chalcone Treatment

The chalcone 2,2′,5′-trihydroxychalcone (225THC) was purchased from Indofine Chemical Co. (Hillsborough, NJ, USA, at 97% purity) and was applied to microglia and neurons to test for any inherent toxicity. 225THC was added at (final concentrations) 1 *μ*M, 5 *μ*M, 10 *μ*M, 25 *μ*M, 50 *μ*M, 100 *μ*M, or 500 *μ*M in 100% sterile DMSO (Sigma D2650; hybri-MAX) as well as a solvent control of 100% DMSO and incubated for 24 h. Live/dead assays were carried out using Hoechst 33342 for total cell number and propidium iodide (PI) for dead cells as previously described [22]. Cells were visualised and images captured with a Zeiss Axioskop 2 fluorescence microscope (Oberkochen, Germany) and images were captured using Zeiss Axiovision Imaging System 4.8 software. The number of live/dead cells was counted manually, or using Image J software. At least 5 fields per coverslip, 3 coverslips per 225THC concentration from 3 separate cell platings were analysed.

### 2.3. 225THC Treatment of Surveillant and Inflammatory Microglia

The effects of 225THC on microglial activation were tested by treating primary microglia or BV2 microglia with 225THC (1 *μ*M, 5 *μ*M, 10 *μ*M, 50 *μ*M, 100 *μ*M, and 500 *μ*M) with or without 2 *μ*g/mL of the Toll-like receptor 4 (TLR4) agonist, lipopolysaccharide (LPS), for 24–48 h. This concentration of LPS was used as this produces maximal iNOS expression as determined in previous experiments and published papers from our laboratory [14, 23]. The cells were subsequently analysed by live/dead assay, Western blot, and cell supernatants analysed for cytokine secretion as described. To test for protective effects of 225THC against neuroinflammation-driven neurotoxicity, CGCs were directly treated with 225THC (5 *μ*M, 50 *μ*M, and 500 *μ*M) and then either 2 *μ*g/mL LPS or 10 ng/mL IFN-*γ* to activate resident microglia in the cultures. Following 24 h, CGC cultures were analysed by Hoechst 33342 staining to assess nuclear morphology as described previously [22].

### 2.4. Western Blot of Inducible Nitric Oxide Synthase Expression

Cells were treated for Western blotting using standard techniques followed by blot visualisation with ECL. Beta- (*β*-) actin was used as a loading control in all gels and protein bands analysed following densitometry with Image J software. Primary antibodies used were anti-iNOS, 1 : 2500, overnight followed by 1 : 5000 HRP conjugated goat anti-rabbit, for 1 h, and anti-*β* actin 1 : 10000 overnight, followed by HRP conjugated goat anti-mouse, 1 : 20,0000 for 1 h. Goat anti-rabbit peroxidase secondary antibody was from Sigma (Poole, UK), donkey anti-goat peroxidase secondary antibody was from GeneTex (Insight Biotech, Wembley UK), goat anti-arginase-1 was from Santa Cruz Biotech (http://www.scbt.com/), and rabbit anti-inducible nitric oxide synthase (iNOS) was from BD Biosciences (http://www.bdbiosciences.com/).

### 2.5. Dihydroethidium Fluorescence Imaging of Superoxide Generation

The superoxide sensitive fluorescent dye dihydroethidium (dHEth) was used to assess microglial superoxide generation and its regulation by the chalcone. Dihydroethidium is oxidised to 2-hydroxyethidium (2-OH-E^+^) upon exposure to superoxide specifically, correlating with a shift in fluorescence from blue to red which is detectable by fluorescence microscopy [24] and we have used this previously to assess superoxide generation in microglia [22]. BV2 microglia were treated with 225THC, LPS, or 10 nM phorbol 12-myristate 13-acetate (PMA) (the latter as a positive control for the generation of superoxide by NADPH activity [22, 25]) for 24 h and then incubated with 5 *μ*M dHEth for 40 min to identify superoxide-producing microglia as described previously [22] plus 0.6 *μ*g/mL Hoechst 33342 for 40 min was used to counterstain all nuclei for total cell number. Microglia were imaged by fluorescence microscopy as above, and superoxide-positive microglia, as indicated by red nuclei, were counted and expressed as a percentage of total cell number. To ensure that the red fluorescence was due to superoxide production, experiments were carried out by incubation of the cells with 10 *μ*M apocynin [22].

### 2.6. ELISA of Secreted Cytokines by Microglia

TNF-*α* or IL-1*β* concentrations in primary rat microglia cell culture supernatants were quantified using Quantikine Rat TNF-*α* or IL-1*β* Immunoassay kit according to the manufacturer's instructions (R&D Systems, Abingdon UK). Cytokine concentrations in cell supernatants were determined against a standard curve of TNF-*α* or IL-1*β*. In addition, a range of inflammatory mediators were analysed in BV2 cell culture supernatants with an Inflammatory ELISA strip assay according to the manufacturer's instructions (Signosis Inc., Caltag MedSystems Ltd., Buckingham, UK). In this case, all values were presented as a percentage of control values. For each condition, the cytokine content in supernatants was analysed from three coverslips of microglia in three independent cell platings with each sample assayed in duplicate. All other chemicals and reagents were from Sigma (Poole, UK).

### 2.7. Flow Cytometric Analysis of Microglial Inflammatory Markers

BV2 cells were treated with 1, 5, or 10 *μ*M of 225THC for 2 hours prior to the addition of 2 *μ*g/mL LPS for a further 48 hours. Cells were harvested by washing in Dulbecco's phosphate buffered saline (PBS, without Ca^2+^ and Mg^2+^) for 20 minutes at 37°C and then resuspended in cold PBS containing 0.5% BSA/0.05% NaN_3_ to metabolically fix the cells. Cells were stained with anti-mouse CD11b-FITC, CD40-FITC, CD54-FITC, and CD68-FITC, or appropriate FITC-conjugated isotype control antibodies (Miltenyi Biotec, UK) as per the manufacturer's instructions and cell surface staining was assessed by flow cytometry (FACSCalibur running CellQuest Pro; Becton Dickinson, UK) and analysed using Flowing software v2.5.1. Data are presented as average fold change in mean fluorescence intensity (MFI) versus untreated cells ± SEM from 4 independent experiments.

### 2.8. Statistical Analysis

Statistical analyses were performed using one-way ANOVA with Tukey* post hoc* analysis, where comparisons were made between treatments and control untreated cells, and also between treatment groups as indicated. Where stated, Student's *t*-test was used and all data were from 3 separate experiments, ^*∗*^ *p* < 0.05, ^*∗∗*^ *p* < 0.01, and ^*∗∗∗*^ *p* < 0.005 compared with controls or as indicated in figures. Where imaging was performed, a minimum of 3 fields of view were analysed from 3 coverslips per condition. Images of fluorescent fields of cells or Western blots are representative of analysed data from 3 independent experiments. The densitometry for Westerns was performed using Image J, measuring the optical density (OD) of each band and normalising to the *β*-actin obtained for that particular sample. For the statistical analysis of these data, each treatment was compared with control untreated cells by ANOVA analysis, and appropriate comparisons were also made between treatment groups ± inhibitors using Student's *t*-test.

## 3. Results and Discussion

### 3.1. Effects of 225THC in Primary Rat Cerebellar Granule Cell Neuronal Cultures

**Figure 1:**
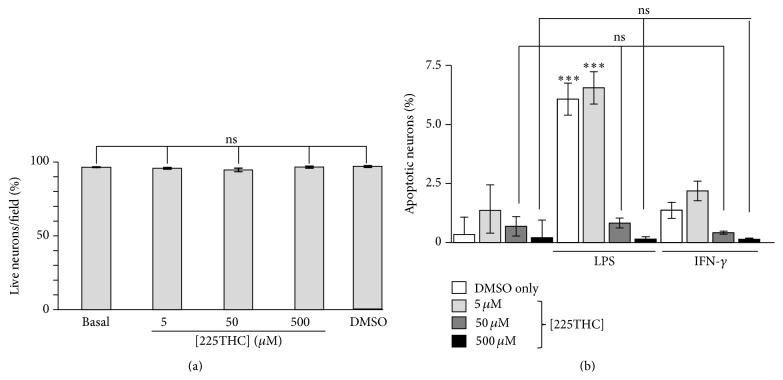
225THC is nontoxic and neuroprotective in stimulated primary rat neuronal cultures (cerebellar granule cell neurons; CGCs). Effects of 225THC on primary rat CGC neurons. (a) Toxicity assay of 225THC on primary rat CGC neurons. Data from three separate experiments represent the mean number of live neurons (as assessed by live/dead assay) present per field of view following incubation for 24 h with different concentrations of 225THC (0, 5, 50, and 500 *μ*M) for 24 h. DMSO is the solvent control; basal is CGCs with nothing added. Statistical analysis was performed by ANOVA with Bonferroni* post hoc* analysis and revealed no toxicity at the concentrations tested when all concentrations were compared with basal. The DMSO solvent control was also not toxic. (b) Direct treatment of CGC cultures for 24 h with 225THC (0, 5, 50, and 500 *μ*M) and 2 *μ*g/mL LPS or 10 ng/mL IFN-*γ* to activate resident microglia in the cultures. The percentage of apoptotic cells following Hoechst 33342 visualisation of nuclear condensation is shown for each treatment. Statistical analysis was performed by a two-way ANOVA with Bonferroni* post hoc* analysis; *∗∗∗* indicates *p* < 0.001; ns, not significant when compared as indicated or with the appropriate basal control.

We initially investigated whether the 225THC was directly toxic to neurons by incubating primary rat cerebellar granule cell (CGC) neuronal culture neurons with concentrations of 5–500 *μ*M 225THC for 24 h. This range of concentrations was assessed as previous studies using similar compounds have identified activity within this range [20, 26, 27]. Microscopic live/dead analyses revealed no significant toxicity in these cultures with DMSO solvent control, or with 5, 50, or 500 *μ*M 225THC (Figure 1(a)) when compared with untreated cells. We next investigated the potential neuroprotective properties of 225THC in these cultures (which we have shown previously contain 2–5% microglia) [28, 29]. A range of 225THC concentrations were preincubated with CGCs for 1 hour, followed by stimulation with 2 *μ*g/mL LPS or 10 ng/mL IFN-*γ* to induce an inflammatory phenotype within the CGC cultures. After 24 h, the CGCs were imaged with Hoechst 33342 to visualise apoptotic nuclei within the cultures. LPS or IFN-*γ* significantly increased the percentage of apoptotic cells in the neuronal cultures above basal (Figure 1(b)) whilst pretreatment with 225THC at 50 or 500 *μ*M significantly reduced the percentage of apoptotic cells in cultures treated with LPS or IFN-*γ*. Since LPS and IFN-*γ* are potent activators of microglia, with no known effects on cerebellar neurons and since incubation with chalcone directly to these cultures, in the absence of inflammation, did not enhance basal levels of neuronal survival, it is likely that suppression of the neuroinflammatory and neurotoxic effects seen here are due to suppression of microglial responses. Thus we determined how these effects might be mediated. One proposed mechanism for the neurotoxicity observed is that activated microglia release proinflammatory cytokines that cause cytotoxicity to the neurons [15, 30, 31].

### 3.2. 225THC Inhibits Proinflammatory Cytokine Secretion by LPS-Stimulated Primary Rat Microglial Cells

**Figure 2:**
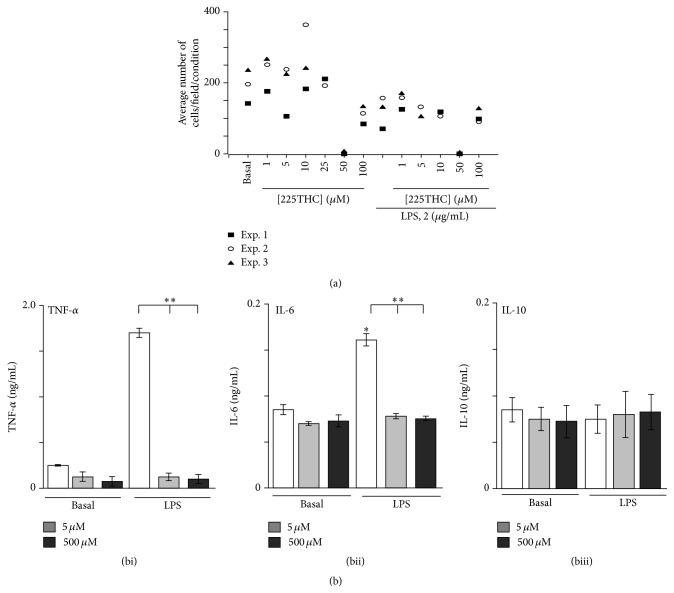
225THC is nontoxic and inhibits inflammatory cytokine responses in stimulated primary rat microglial cultures. Effects of 225THC on primary rat microglial cells. (a) Toxicity assay of 225THC on primary cultured rat microglia. Data shown from three separate experiments represent the mean number of live primary microglia (as assessed by live/dead assay) present per field of view following incubation for 24 h with different concentrations of 225THC (0, 1, 5, 10, 50, and 100 *μ*M) for 24 h with or without coactivation of the microglia with LPS (2 *μ*g/mL). (b) ELISA assessment of secreted cytokines in LPS (2 *μ*g/mL) activated primary rat microglia cultures treated with 225THC at the concentrations indicated. Statistical analysis was performed by a two-way ANOVA with Bonferroni* post hoc* analysis; *∗* indicates *p* < 0.05, ^*∗∗*^ *p* < 0.01; when compared with basla or as indicated.

As a preliminary assessment, we again investigated the potential cellular toxicity of 225THC when added to primary rat microglial cultures. We observed that, in three separate experiments, a specific concentration of 50 *μ*M 225THC appeared to induce significant cell loss from coverslips in both nonstimulated and LPS-stimulated cells, whilst concentrations above and below this did not (Figure 2(a)). This was most likely due to loss of contact rather than toxicity as there was no increase in PI staining. DMSO controls did not affect cell numbers (data not shown).

We next assessed cytokine secretion in microglial cell culture supernatants under basal (nonstimulating conditions) and following LPS stimulation (2 *μ*g/mL) in the presence of 50 or 500 *μ*M 225THC (Figure 2(b)). The basal secretion of TNF-*α*, IL-6, and IL-10 was not significantly modulated by 5 *μ*M or 500 *μ*M 225THC whilst LPS-evoked TNF-*α* and IL-6 secretion, but not IL-10, was significantly inhibited by 5 *μ*M or 500 *μ*M 225THC (Figures 2(bi), 2(bii), and 2(biii), resp.).

### 3.3. 225THC Modulates Inflammatory Cytokine and Chemokine Responses in Murine BV2 Microglial Cells

**Figure 3:**
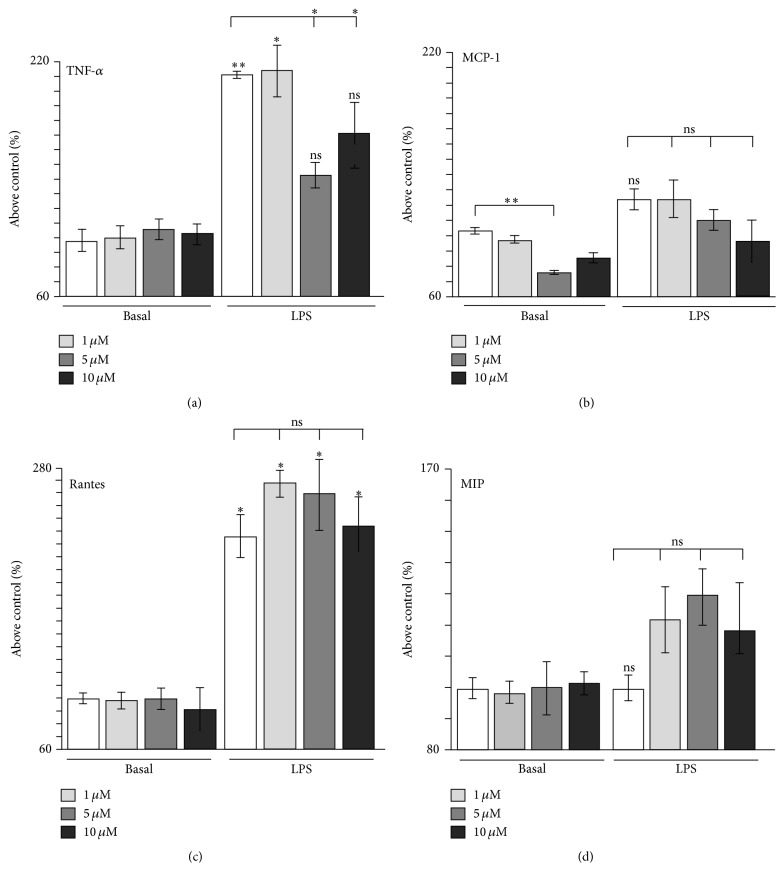
225THC modulates inflammatory cytokine and chemokine responses in murine BV2 microglial cells. 225THC-modulation of cytokine/chemokine secretion by the BV2 microglial cell line. ELISA assessment of cytokine ((a) TNF-*α*) and chemokine ((b) MCP-1; (c) RANTES; (d) MIP) secretion by BV2 microglial cells following stimulation with LPS (2 *μ*g/mL) for 24 h in the presence of 225THC at the concentrations indicated. Statistical analysis was performed by a two-way ANOVA with Bonferroni* post hoc* analysis; *∗* indicates *p* < 0.05, ^*∗∗*^ *p* < 0.01; ns, not significant when compared with basal or as indicated.

To investigate the microglial aspects further, we used the BV2 mouse microglial cell line which is a well validated substitute for primary microglia [32]. Further examination of a lower range of 225THC concentrations (1 *μ*M, 5 *μ*M, and 10 *μ*M) following a 48 h LPS stimulation revealed selective inhibition of LPS-induced TNF-*α*, in line with our findings in primary microglia (Figure 3(a)). In addition, the basal secretion of monocyte chemoattractant protein-1 (MCP-1) was also significantly inhibited (Figure 3(b)). We did not observe modulation of basal or LPS-evoked secretion of IFN-R, IL-1*α*, IL-1*β*, (*data not shown*) or of RANTES (Figure 3(c)) or MIP (Figure 3(d)). These data suggest that 225THC has an effect on the secretion of inflammatory cytokines such as TNF-*α*, IL-6, and MCP-1, and the reduction of these cytokines may aid neuroprotection. Interestingly, in line with our findings, Herencia et al. [26] report that the chalcone used in their study inhibited TNF-*α* mouse air pouch tissue levels from leukocytes stimulated by zymosan, whilst Bano et al. [33] reported suppression of LPS-evoked TNF-*α* secretion from a mouse macrophage line with a synthetic chalcone. In addition, we found 225THC inhibited the secretion of two other cytokines, IL-6 and MCP-1. IL-6 has well recognised roles in neuroinflammation and promotes microgliosis [34] whilst MCP-1 (also known as CCL-2) acts as a chemoattractant to recruit monocytes and macrophages into the brain [35]. Interestingly, recent work suggests different chalcones may selectively inhibit the secretion of different groups of cytokines and inflammatory mediators [27]. We did not see significant modulation by 225THC of RANTES, MIP, or IL-10. Other chalcones have been shown to promote the production of neurotrophic factors such as GDNF from astrocytes [20] and this is worth future exploration for microglia.

### 3.4. Modulation of iNOS Expression and ROS Generation by 225THC

**Figure 4:**
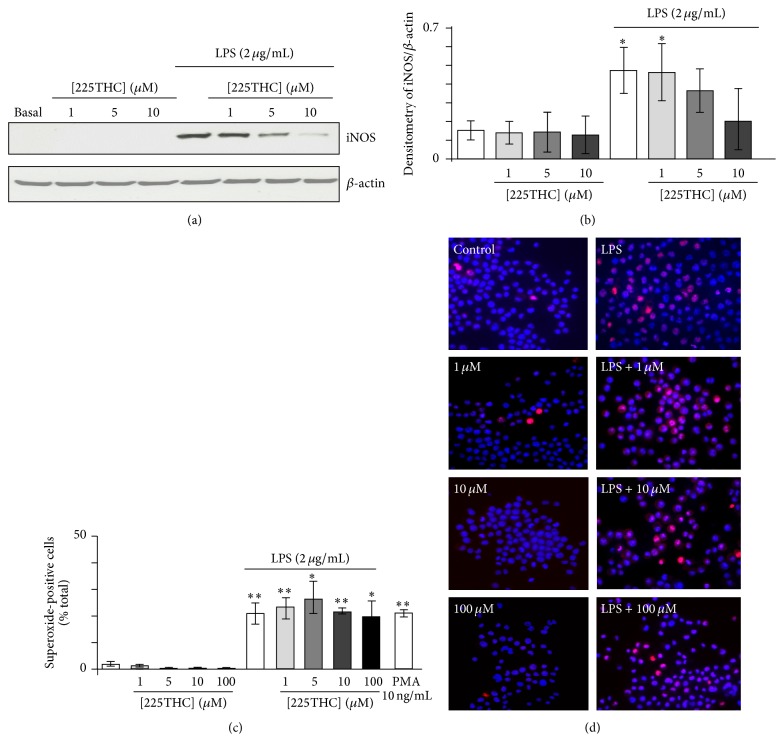
225THC inhibits the LPS-induced expression of iNOS, but not NADPH-driven superoxide production, in BV2 microglia. Modulation of BV2 iNOS expression, but not superoxide production, by 225THC. Representative Western blots of iNOS expression and *β*-actin as a loading control (a) and densitometry (b) of three separate blots for BV2 microglia exposed to 225THC at the stated concentrations ± LPS (2 *μ*g/mL) for 24 h. Statistical analysis was performed by a two-way ANOVA with Bonferroni* post hoc* analysis; *∗* indicates *p* < 0.05 when compared with basal or as indicated. (c) Superoxide generation in BV2 microglia measured by dihydroethidium fluorescence following 24 h exposure to 225THC at the concentrations indicated ± LPS (2 *μ*g/mL). The phorbol, PMA, was used as a positive activator of superoxide generation. (d) Representative fields of BV2 microglia generating intracellular superoxide following 24 h exposure to 225THC +/− LPS. Statistical analysis was performed by a two-way ANOVA with Bonferroni* post hoc* analysis; *∗* indicates *p* < 0.05; *∗∗* indicates *p* < 0.01 when compared with basal or as indicated.

The expression of iNOS was assessed in BV2 microglia under basal conditions and following LPS stimulation (2 *μ*g/mL) for 48 h in the presence of 225THC (1 *μ*M, 5 *μ*M, or 10 *μ*M). No obvious effects were observed on the expression of iNOS in nonstimulated microglia; that is, 225THC did not induce expression of iNOS (Figure 4(a)). However LPS-evoked stimulation of iNOS expression was significantly inhibited by 225THC at concentrations above 5 *μ*M (Figures 4(a) and 4(b)). We next investigated microglial superoxide generation using microscopic fluorescence imaging (Figure 4(c)). No obvious effects of the 225THC were observed on the basal cell numbers expressing superoxide-positive cell nuclei and whilst the number of superoxide-positive cells was increased following LPS stimulation this was not significantly regulated by 225THC concentrations from 1 to 100 *μ*M (Figure 4(c)) or at 500 *μ*M (*data not shown*). The positive control of NADPH oxidase activation, PMA (10 ng/mL), induced a significant increase in superoxide-positive cells, in line with previous findings [22].

The inhibition of iNOS expression in microglia is in agreement with findings for another chalcone, 4-dimethylamino-3′,4′-dimethoxychalcone, on mouse macrophages stimulated with zymosan [22]. This chalcone inhibited iNOS expression with a similar concentration (~10 *μ*M) to the chalcone used in the present study. Herencia et al. [26] proposed that the effect on iNOS expression was via upstream modulation of NADPH oxidase-mediated intracellular signalling. Since we did not detect modulation of microglial superoxide generation by 225THC, it seems unlikely that this particular chalcone possesses this mechanism of action. Other chalcones have also been reported to modulate nitric oxide production, or iNOS expression following LPS stimulation of a mouse macrophage cell line [36], the BV2 microglial cell line [27], and a rat microglial cell line [37] within similar compound concentration ranges of 1–50 *μ*M. The exact signalling pathways inhibited by different chalcones may be diverse, as some studies suggest modulation of nuclear factor-*κ*B, MAPK, or c-Jun pathways and others suggest STAT1 pathways may be involved [26, 37].

### 3.5. Modulation of Microglial Inflammatory Phenotype

**Figure 5:**
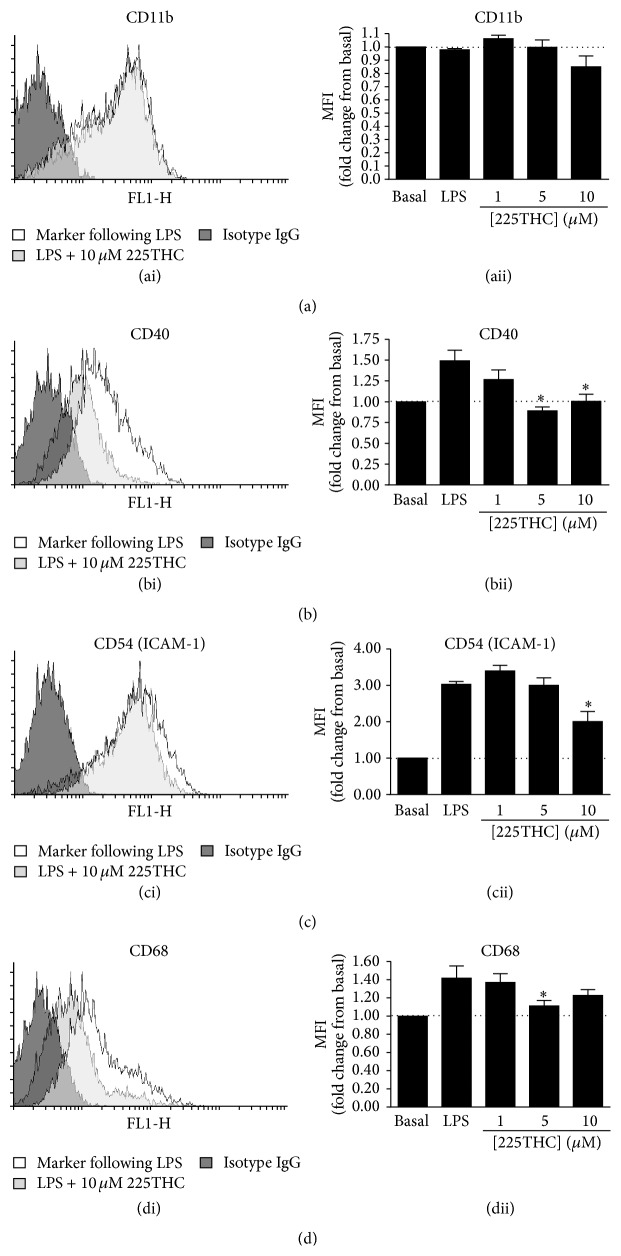
225THC treatment modulates inflammatory cell surface marker expression in LPS-treated BV2 microglia. Flow cytometric analysis of 225THC-treated BV2 microglia following LPS stimulation. (a) Representative histogram plots of BV2 cell surface CD11b, CD40, CD54, and CD68 expression following 2 *μ*g/mL LPS-treatment for 48 hours (unfilled histograms), versus isotype control (black-filled histograms), and in the presence of 10 *μ*M 225THC (grey-filled histograms). (b) Summary data showing fold changes in CD11b, CD40, CD54, or CD68 expression by mean fluorescence intensity (MFI) from 4 independent experiments. Data are mean values ± SEM where *∗* indicates *p* < 0.05 by Student's *t*-test.

Upon LPS stimulation, microglia adopt an inflammatory phenotype which is characterised by enhanced cell surface expression of key adhesion (CD11b, CD54), and costimulatory molecules (CD40). We investigated the effects of 225THC treatment on BV2 cell surface expression of CD11b, CD40, CD54, and CD68 in basal and LPS-treated BV2 microglia (Figure 5). Flow cytometric analysis (FACs) of marker mean fluorescence intensity (MFI) revealed no significant changes in basal CD40, CD54, CD68, or CD11b expression levels following 225THC treatment for 48 h (data not shown). However, following stimulation with 2 *μ*g/mL LPS, CD40 (Figure 5(bii)), CD54 (Figure 5(cii)), and CD68 (Figure 5(dii)) were significantly upregulated and these changes in expression were abrogated in the presence of 5 or 10 *μ*M 225THC. CD40 appeared to be the most potently regulated marker, with both 5 and 10 *μ*M 225THC completely inhibiting LPS-induced upregulation (Figure 5(bii)) while the treatment had more moderate effects on CD54 and CD68 marker expression. As in the basal state, 225THC had no effect on CD11b expression by LPS-stimulated microglia (Figure 5(aii)).

Overall, these data suggest that 225THC targets the signalling pathways downstream of LPS binding and abrogates the expression of genes such as TNF-*α*, iNOS, and CD40. Since these are all reported NF-*κ*B driven genes, future work could investigate this further to identify the exact pathways modified by 225THC. Abrogating this gene expression produced a less neurotoxic microglial phenotype which is likely to be beneficial for a number of neurodegenerative diseases in which aberrant microglial inflammatory reactivity is implicated. Future work could pinpoint further the microglial phenotype favoured by 225THC exposure and explore the genotype of the microglia.

## Abbreviations

ANOVA: Analysis of variance
DPPH: 2,2-Diphenyl-1-picrylhydrazyl
dHEth: Dihydroethidium
CGCs: Cerebellar granule neurons
DIV: Days* in vitro*
DAPI: 4′,6-Diamidino-2-phenylindole
DMSO: Dimethylsulphoxide
ECL: Electrochemiluminescence
ELISA: Enzyme-linked immunosorbent assay
GABA_A_: *γ*-Aminobutyric acid
IFN-*γ*: Interferon-*γ*
IL-1*β*: Interleukin-1*β*
IL-6: Interleukin-6
IL-10: Interleukin-10
iNOS: Inducible nitric oxide synthase
LPS: Lipopolysaccharide
MCP-1: CCL2/monocyte chemoattractant protein-1
MEM: Modified Eagle's medium
MIP: Macrophage inhibitory protein
MCP-1: Monocyte chemoattractant protein-1
NGS: Normal goat serum
PBS: Phosphate buffered saline
PFA: Paraformaldehyde
2-OH-E^+^: 2-Hydroxyethidium
RANTES: CCL5/regulation on activation normal T cell expressed and secreted
ROS: Reactive oxygen species
TNF-*α*: Tumour necrosis factor-*α*.
